# The immune response and aging in chronic inflammatory demyelinating polyradiculoneuropathy

**DOI:** 10.1186/s12974-021-02113-2

**Published:** 2021-03-22

**Authors:** Kathleen M. Hagen, Shalina S. Ousman

**Affiliations:** 1 Department of Neuroscience, Hotchkiss Brain Institute, University of Calgary, Calgary, AB T2N 4N1 Canada; 2 Departments of Clinical Neurosciences and Cell Biology and Anatomy, Hotchkiss Brain Institute, University of Calgary, Calgary, AB T2N 4N1 Canada

**Keywords:** Peripheral nervous system, Chronic inflammatory demyelinating polyradiculoneuropathy, Immune system, Aging, Immunosenescence

## Abstract

Chronic inflammatory demyelinating polyradiculoneuropathy (CIDP) consists of various autoimmune subtypes in which the peripheral nervous system (PNS) is attacked. CIDP can follow a relapsing-remitting or progressive course where the resultant demyelination caused by immune cells (e.g., T cells, macrophages) and antibodies can lead to disability in patients. Importantly, the age of CIDP patients has a role in their symptomology and specific variants have been associated with differing ages of onset. Furthermore, older patients have a decreased frequency of functional recovery after CIDP insult. This may be related to perturbations in immune cell populations that could exacerbate the disease with increasing age. In the present review, the immune profile of typical CIDP will be discussed followed by inferences into the potential role of relevant aging immune cell populations. Atypical variants will also be briefly reviewed followed by an examination of the available studies on the immunology underlying them.

## Background

In 1958, Austin published a review on several cases of recurrent demyelinating polyneuropathy, now termed CIDP [1]. Broadly speaking, CIDP is an immune-mediated demyelinating neuropathy that is preceded by a chronic progressive or relapsing/remitting course [2]. It is worth noting however, that CIDP is a very heterogeneous disorder with typical and atypical variants [3] (Table 1).

**Table 1:** CIDP variants and their main features

CIDP variant	Main features
**Typical CIDP**	• Symmetric, sensory and motor symptomology following a chronic course • A subpopulation has an acute onset
**Sensory predominant CIDP**	• Involves sensory over motor symptoms following a chronic course
• **CISP**	• Younger age of onset
**Seropositive CIDP**	• Presence of antibodies against NF155, tremors, sensory and cerebellar ataxia, weakness
• **NF155**	• Younger age of onset
• **NF186**	• Presence of antibodies against NF186, subacute onset, sensory ataxia
• **CNTN1**	• Presence of antibodies against NF186, subacute onset, sensory ataxia • Older age of onset
• **CASPR**	• Presence of antibodies against Caspr, subacute and severe, motor dominant, painful
**Lewis-Sumner Syndrome** (aka multifocal-acquired demyelinating sensory and motor polyneuropathy)	• Asymmetric sensory and motor symptoms following a chronic course
**Motor predominant CIDP**	• Involves motor over sensory symptoms following a chronic course • More common in juveniles
**Distal-acquired demyelinating symmetric neuropathy (DADS)**	• Symptoms appear distally and are predominantly sensory over motor following a chronic course

In a meta-analysis, it was concluded that the pooled incidence rate of CIDP is 0.33–2.81 per 100,000 person-years [4]. In general, CIDP affects more men than women and is more common in those over 50 years of age [2]. It has been reported that approximately 50% of patients have a “typical” disease course which is defined as progressive, symmetric sensory-motor weakness where the progressive phase lasts a minimum of 2 months [3]. There is also a phenotype with an acute onset which accounts for approximately 18% of cases; these patients reach peak disease within 2 months of diagnosis followed by progression or a relapsing-remitting course.

Along with typical CIDP, there are also many atypical and less common variants. Atypical variants include sensory predominant, seropositive (i.e., positive for antibodies), Lewis-Sumner syndrome (LSS; aka multifocal acquired demyelinating sensory and motor polyneuropathy, MADSAM), motor predominant, and distal acquired demyelinating symmetric neuropathy (DADS) [3, 5]. In addition, a portion of atypical CIDP patients do progress into a typical disease form; however, this is associated with a longer disease course [5] (Table 1). Typical CIDP as well as the aforementioned atypical variants will be discussed further in the present review.

## Typical CIDP

As mentioned above, typical CIDP is a chronic progressive neuropathy that can affect motor and sensory functions and accounts for approximately 50% of CIDP cases [3]. In ultrasound studies, it has been shown that the nerves of patients are swollen and at disease onset the enlargement of the nerves is more proximal. As the disease becomes more chronic, the enlargement of the nerves is more generalized [6, 7]. Hypomyelination, onion bulb formation, abnormal Schwann cell morphology, and instances of irregular paranodal loops are key morphological aspects of typical CIDP [7, 8]. However, hypertrophy of cervical roots, brachial plexus, and/or lumbar plexus have also been reported in some patients [9]. In addition to these morphological changes, there is abnormal staining for contactin-associated protein 1 (Caspr) and voltage-gated sodium (Nav) channel. More specifically, diffuse puncta of varying size are stained along the length of the axon while within the actual nodal regions, staining is similar to controls. Finally, there is a notable increase in macrophage clusters around endoneurial blood vessels in CIDP patients compared to healthy controls and hereditary neuropathies [10].

There are very few studies examining the role of the aging immune system in the context of CIDP despite the knowledge that the age of onset of patients with CIDP has been found to impact the symptomology of their disease [11]. Specifically, in juveniles (2–20 years old), approximately half had a subacute progression while in adults (21–64 years old), and the elderly (65–90 years old), a chronic insidious progression was more common (i.e., 88% and 91%, respectively). Further, relapses and motor dominant CIDP are more common in juveniles but the frequency of these decreases with increasing age. Of further note, subperineurial edema was seen in both juveniles and adults (with both higher than in the elderly), although there were no significant differences in the composition of the inflammatory infiltrate between groups. Of all the groups, functional recovery was least frequent in the elderly group. Moreover, patients with a younger age of onset tended to have a better disease outcome and less disease-related death [2].

### Cellular milieu

#### Schwann cells

Joshi and colleagues have demonstrated that the ability of Schwann cells to contribute to regeneration of damaged nerves in CIDP patients is impaired [12]. In order to study this, cultured Schwann cells were exposed to sera from CIDP patients or controls and then transplanted into nerves. Relative to control-treated Schwann cells, both human and rat Schwann cells exposed to CIDP patient sera displayed decreased p57kip2, c-Jun, brain-derived neurotrophic factor (BDNF), and glial cell line-derived neurotrophic factor (GDNF) mRNA expression, an upregulation of nerve growth factor (NGF) mRNA, and a reduction in granulocyte-macrophage colony-stimulating factor (GM-CSF). When GM-CSF was used to treat CIDP-patient sera-exposed Schwann cells, the cytokine rescued the levels of p57kip2, c-Jun, BDNF, and GDNF mRNA expression and led to an increase in NGF mRNA above both CIDP patient sera-treated and control-treated Schwann cells. From this, the authors concluded that CIDP detrimentally affects the ability of Schwann cells to be pro-regenerative and that GM-CSF may be a key factor in this abnormal functioning.

#### Immune cells

A hallmark of typical CIDP is the breakdown of myelin. It has been reported that this is primarily caused by macrophages entering into the nerve in a localized area and damaging the myelin [13]. Lymphocytes have also been observed in the endoneurium in contact with macrophages or other lymphocytes [13]. Schmidt and colleagues noted that in sural nerve biopsies of CIDP patients there was an elevated presence of epi- and endoneurial T cells and macrophages [14]. Active CIDP sural nerve biopsies have also revealed an increased density of major histocompatibility complex (MHC) class II positive staining in the perineurium and epineurium compared to controls, particularly by endoneurial endothelial cells [15].

Between typical and atypical CIDP variants, there are differences in the underlying T cell response [16]. In a study by Staudt and colleagues, they found that atypical CIDP patients had an insignificant trend toward an increased T cell response to myelin antigens compared to typical CIDP patients. Atypical CIDP patients did have significantly higher numbers of T cells and specifically CD4^+^ T cells than typical CIDP patients. Moreover, there was an insignificant trend towards increased numbers of CD8^+^ effector memory and CD8^+^ central memory T cells in atypical versus typical CIDP in this study; in this study however, the authors did not further differentiate between atypical subtypes.

Towards identifying the contribution of CD8^+^ T cells in the pathogenesis of CIDP, Schneider-Hohendorf et al., examined the T cell receptor repertoire in CIDP patient blood and biopsy tissue [17]. They found that CD8^+^ T cells are clonally expanded in CIDP with a restricted Vβ repertoire. Mausberg and colleagues have also examined the T cell receptor repertoire in CIDP patients [18]. In their study, they used CDR3 spectratyping to map individual T cell repertoires and found that untreated CIDP patients tended to have distorted CD8^+^ T cell receptor repertoires. The authors also noted that intravenous immunoglobulin (IVIg) treatment normalized the T cell repertoire of CIDP patients. In keeping with these findings, Matsumuro and colleagues examined the histology of sural nerve biopsies and found an increased presence of CD8^+^ T cells over CD4^+^ [19]. In addition, they reported active demyelination in the presence of human leukocyte antigen (HLA)-DR-positive macrophages and areas of remyelination where HLA-DR-positive Schwann cells were seen. Importantly, they noted that demyelination correlated with T cell presence in the nerves of CIDP patients.

With respect to functional CD4^+^ T cell subtypes, it has been found in active CIDP patients that there is an increased frequency of T helper (Th)17 cells and Th1/Th17 cells but no changes in Th1 cell abundance in the peripheral blood compared to remitting CIDP and control groups [20]. Further, when active CIDP patients were monitored longitudinally into remission, a decrease in Th17 and Th1/Th17 cells was seen over time. In the cerebrospinal fluid (CSF), there was a higher frequency of Th1, Th17, and Th1/Th17 cells in active CIDP compared to remitting CIDP and control groups. However, when patients with active CIDP were examined into remission, there were only significant reductions in the CSF for Th17 and Th1/Th17 cells. Further, interleukin (IL)-17 levels in serum and expression of RORγt were both elevated in active CIDP compared to remitting CIDP and controls. Because of this data showing that Th1 and Th17 cells may be involved in CIDP, Horiuchi and colleagues determined the intracellular interferon (IFN)-γ/IL-4 ratio in CD4^+^ cells in order to assess if Th2 cells participated in CIDP [21]. They found that in CIDP patients, there was a higher percentage of IL-4^+^IFN-γ^-^ cells compared to controls which would be indicative of an increase in Th2 cells. However, it is worth noting that this study did not specify the phase of disease or treatments received, if any, of CIDP patients which may have an impact on the T cell profile.

Besides CD4^+^ and CD8^+^ T cells, other immune cell subsets have been identified. Winer and colleagues performed histological evaluations of biopsy tissue and found γδ T cells in 14 out of 20 CIDP patients; it is worth noting that this population was not highly prevalent but was present in 14 different patients [22]. With respect to other immune cell populations, Sanvito and colleagues found no differences among CIDP patients, healthy controls, and other neuropathy patients in total circulating B and T cells and in CD4^+^, CD8^+^ T cells, the ratio of CD4^+^/CD8^+^ T cells, regulatory T cell (Tregs), effector memory T cells, natural killer (NK) T cells, or central memory T cells [23]. They did find differences in NK cells and monocytes, namely, that there were more and less monocytes and NK cells, respectively, in CIDP patients compared to healthy controls. Further, in T cell suppression assays, there was a smaller mean suppression percentage in CIDP patients compared to healthy controls. Of the 22 CIDP patients enrolled in the study, however, 11 of them were diagnosed with an atypical form of the disease which could have affected the resulting immune populations.

In contrast to the findings of Sanvito and colleagues, Chi and colleagues have shown that there is a significant decrease in Treg numbers and a decline in their suppressive ability in CIDP patients particularly in the progressive and relapse phases compared to healthy controls [24]. That is, non-specific suppressor T cell functioning was found to be impaired in a concanavalin A assay using peripheral blood mononuclear cells (PBMCs) from CIDP patients [25]. However, following treatment with prednisone or plasma exchange, suppressive functions improved [26]. Also, in an examination of NK-T cell populations between CIDP and multiple sclerosis patients, it was found that in multiple sclerosis patients there was a reduction in the presence of Vα24JαQ NK-T cells while in CIDP, there was a marked infiltration of this cell type along with the presence of IL-4 mRNA [27]; this is noteworthy because NK-T cells are potent cytokine producers.

##### Evidence for a role for aging T cells

**Fig. 1:**
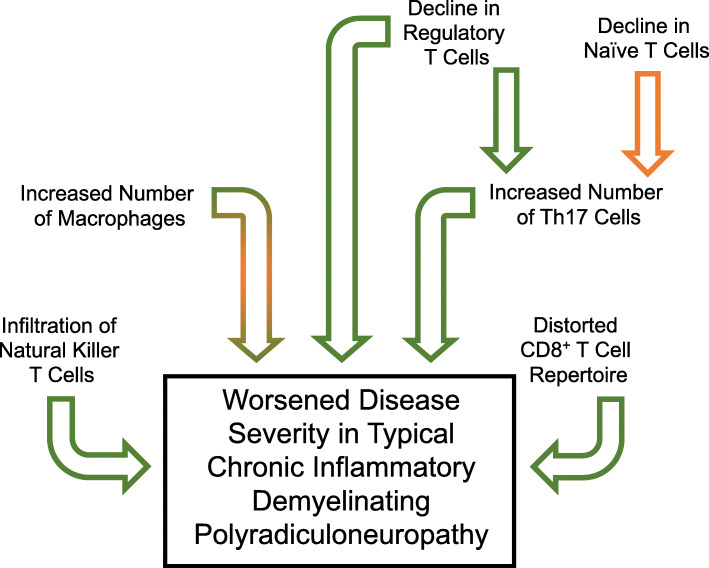
Proposed mechanism of age and immunological contributions to typical CIDP pathology. In CIDP, there is a decline in regulatory T cells which are opposite to the age-related increase in these cells. In combination with a decline in naïve T cells with age, this may contribute to the CIDP-related increase in Th17 cells, thus creating an imbalance in Tregs and Th17 cells. Due to dysregulation of the immune system, this may allow for further pathological contributions of the infiltrating natural killer T cells and distorted CD8+ T cell repertoire seen in CIDP. Also, with age, there is an increase in macrophages within the peripheral nerve and in combination with an increase in macrophages due to CIDP, and this could contribute to an age-related increase in disease severity. Orange arrows = age-related contribution; green arrows = disease-related contribution; combination of green and orange arrows = cumulative contribution of age and disease

In CIDP, Chi and colleagues reported a decrease in Treg numbers [24], while Sanvito and colleagues found no difference in this population. However, as noted above, half of their CIDP patient population was of the atypical variety which may have altered the results of their immune profiling [23]. If there is indeed a reduction in Tregs with CIDP, this may leave the patient open to an unregulated insult by other immune cells. In aging research, several groups have reported an increase in Treg frequency with age [28–30]; therefore, if there is no longer an increase in these cells with age due to CIDP, this could have further unknown, likely negative, consequences in these patients (See Fig. 1).

In addition to Treg alterations, there was an increase in Th17 cells in active CIDP but a decrease during remission [20]. This study also found an increase in the Th1/Th17 ratio during active disease and a decrease in the ratio during remission. Changes in these CD4^+^ T cell populations may be due to an age-related decline in naïve T cells and a change in the balance of Tregs to Th17 cells [31–33]. Such alterations would diminish immunoregulatory mechanisms, thus allowing for repeated autoimmune insults (Fig. 1).

Some groups have reported an increase in CD8^+^ T cells in CIDP [17, 19] while Sanvito and colleagues reported no difference relative to controls [23]. With age, there is a decline in both naïve and central memory CD8^+^ T cells [34]; however, others have reported an increase in memory CD8^+^ T cells [35]. CD8^+^ T cells undergo phenotypic change with senescence (i.e., decreased CD28 expression) similar to that of CD4^+^ T cells. However, the effect is more frequent and occurs more rapidly in CD8^+^ T cells. In a recent study by Márquez and colleagues, changes in the immune system with age and sex were examined [36]. They reported a significant decline in the percentage of CD8^+^ T cells in PBMCs with age, particularly in males; however, the authors also found an upregulation in genes associated with cytotoxic functions that they attributed to NK cells and memory CD8^+^ T cells with increasing age that was shared between sexes. Finally, in a recent review of aging CD8^+^ T cells, Jergović and colleagues concluded that there may be an underlying disorganization of intrinsic and extrinsic interactions with T cells that are overshadowing a lack of direct age-related declines in CD8^+^ T cell functioning [37]. This potential revelation in combination with decreased Tregs in CIDP and age-related increases in cytotoxicity and memory CD8^+^ T cells may function together with activated macrophages to lead to damage and demyelination within peripheral nerves.

##### Evidence for a role for aging macrophages

As mentioned above, in CIDP there is an increase in macrophage clustering around endoneurial blood vessels [10], as well as an increase in tumor necrosis factor (TNF)-α staining in macrophages located in close proximity to myelinated fibers (the latter will be discussed later in the article) [38]. Macrophages have also been seen damaging myelin in CIDP and therefore are severely implicated in demyelination [13]. Some direct evidence that age may impact macrophage involvement was provided by Shy and colleagues who found that mice heterozygous for myelin protein zero (P0) developed a CIDP-like phenotype that was worse in older mice [39]. The effect was associated with an increased presence of macrophages stripping away myelin. Although there was an increased presence of macrophages in the aforementioned paper, this may be due in part to aging, in addition to a pathological mechanism(s), since with increasing age there is an increase in the number of macrophages within peripheral nerves [40]. In addition, aged macrophages tend to exhibit more pro-inflammatory actions [41] which may further contribute to the worsened phenotype with age. This increased presence and pro-inflammatory activity of the macrophages with age overlaps with what is seen of macrophages in typical CIDP (Fig. 1).

##### Immune cell genetic alterations in CIDP

Compared to healthy controls, CIDP patients display an increase in frequencies of hypoxanthine-guanine phosphoribosyltransferase (HPRT) mutant T cells during the progressive phase of the illness; these mutations can occur during clonal expansion [42]. Further, using T cell clones from one CIDP patient, it was determined that the majority of wild-type and mutant clones were CD4^+^. Taylor and Hughes have gone on to show that there is an increase in activated HLA-DR-positive T cells in CIDP patients relative to controls [43]. Others have also shown an association between CIDP and a genotypic variant on the SH2D2A gene, GA13-16 homozygote [44]. The SH2D2A gene is responsible for the production of T cell-specific adapter protein (TSAd) involved in control of T cell activation. Thus, a mutation could disrupt the homeostatic function of controlling and eliminating T cells that may be reactive to PNS components.

In addition to the SH2D2A variant, genes with the following functions were found to be differentially expressed in skin punch biopsies of CIDP patients compared to controls: regulation of immune responses, chemokines, cell adhesion, transport, protein synthesis, cytoskeletal structure, extracellular matrix components, and proliferation [45]. In terms of specific genes identified, the authors noted that in CIDP patients there was a downregulation of HLA-DPA1, HLA-DPB1, and HLA-DQB2 which are members of the MHC class II family. In contrast, there was an upregulation of lymphatic vessel endothelial hyaluronan receptor, Toll-like receptor 4, Tenascin C, alpha-2-macroglobulin, platelet-derived growth factor-D, caveolin2, endothelial nitric oxide synthase trafficking inducer, and kinase insert domain receptor which encodes the vascular endothelial growth factor receptor. For a more in-depth review of genetic studies in CIDP, please see Blum and McCombe [46].

##### Antigen reactivity of immune cells in CIDP

Khalili-Shirazi and colleagues have demonstrated that a portion of CIDP patients (i.e., 5/13 patients in their study) have T cell responses to P2 antigens [47]. A T cell response to peripheral myelin protein (PMP)-22 (aa 51-64) has also been found in CIDP patients that correlated with an increased spontaneous secretion of IFN-γ and IL-5 compared to healthy controls and other non-immune mediated neuropathies [48]. It is likely that other antigens exist whether they be primary or revealed by epitope spreading.

##### Effects of therapy on immune responses *T cells*

IVIg therapy is the most widely used treatment for CIDP and has been shown to affect the frequency and expression of activation markers in multiple immune cell populations. In one study, it was found that between responders and non-responders to IVIg therapy, there were differences in T cells [49]. Specifically, responders to treatment displayed significantly greater T cell responses against myelin proteins PMP-22 and P2 compared to non-responders at baseline prior to IVIg treatment. The study also revealed that responders had an increased frequency of CD8^+^ effector memory T cells compared to non-responders. Further, in the responders between baseline and follow-up after IVIg treatment, there was a reduction in CD8^+^ effector memory T cells, but no difference in CD4^+^ T cell subsets.

##### *B cells*

In addition to T cells, IVIg treatment has also been found to impact B cells. Normally, naïve and memory B cells have been shown to display reduced inhibitory FcγRIIB on the cell surface of CIDP patients compared to healthy controls; with a greater reduction in the CD19^+^CD27^+^ memory B cells compared to naive [50]. Furthermore, in healthy controls, there was an increase in FcγRIIB expression as B cells transitioned from naïve to memory, but the difference was not significant in CIDP samples. Interestingly, following IVIg treatment FcγRIIB expression increased on naïve and memory B cells, with expression also seen on monocytes in most patient samples. In exploring the underlying disease-mediated mechanism that caused FcγRIIB dysregulation, the authors examined single nucleotide polymorphisms on the FcγRIIB promotor and found that 43% of their CIDP samples were heterozygous for a 386C/120A variant on the promotor whereas <5% of healthy controls possessed this polymorphism. In a similar study by Quast and colleagues, CIDP patients were found to possess decreased mean fluorescence intensity of FcγRIIB on both naïve and memory B cells and CD14^high^CD16^-^ monocytes compared to controls [51]. The CIDP patients also had increased mean fluorescence intensity of FcγRI on both CD14^high^CD16^-^ and CD14^low^CD16^+^ monocytes and increased FcγRIIA on CD14^low^CD16^+^ monocytes compared to controls. Two weeks following IVIg treatment, FcγRIIB surface expression was significantly increased on both naïve and memory B cells and after 4–8 weeks, the expression was maintained. Lastly, FcγRI on CD14^low^CD16^+^ monocytes decreased at 2 weeks post-IVIg, but at 4–8 weeks, expression was not significantly different from pre-treatment.

In addition to B cell numbers and surface markers, IVIg has also been shown to impact B cell cytokines. The cytokine B cell activating factor (BAFF) is elevated in the sera of CIDP patients relative to controls [52] and IVIg treatment has been shown to decrease its levels. Towards identifying the mechanism behind this, Ritter and colleagues found that IVIg did not alter BAFF production but instead that IVIg contains anti-BAFF antibodies that alter serum BAFF concentrations.

##### *Macrophages*

Créange and colleagues have also examined the impact of IVIg treatment on immune cells [53]. Prior to treatment, they found that patients had decreased CD45^+^ populations, particularly CD3^+^CD11a^+^ and CD14^+^CD32^+^ monocytes compared to controls. Immediately after IVIg therapy, there was no change in these populations; however, a week later, there was an increase in CD45^+^, CD3^+^, and CD14^+^ cells approaching control levels. Also, immediately after IVIg, there was a decrease in ICAM-1 expressing T cells which rebounded at 1-week follow-up. Additionally, at 1-week post-IVIg, there was an increase in the number of FcγIIR (CD32^+^)-expressing monocytes but no change in FcγIIIR (CD16^+^) expression.

With respect to macrophage secretory factors, CIDP patients were treated with IVIg and evaluated for serum levels of macrophage colony-stimulating factor (M-CSF) and monocyte chemoattractant protein-1 (MCP-1) [54]. It was found that 1 day after treatment, M-CSF and MCP-1 levels were significantly increased and then rapidly dropped to baseline levels. When examined by response to IVIg, responders at day 1 had significantly higher levels of M-CSF and MCP-1 than non-responders. The findings of this study indicate a possible role of macrophages in IVIg treatment.

##### *NK cells*

The impact of IVIg on NK cells has been studied. Bohn and colleagues examined the impact of IVIg on Fc receptors in NK cells in CIDP patients [55]. They found that treatment led to a decrease in the percentage of NK cells in PBMCs and that antibody-dependent cell-mediated cytotoxicity and NK cytotoxicity were significantly reduced following IVIg. IVIg also led to an increase in IgG binding to NK cells in CIDP patients and a decrease in total numbers of lymphocytes and CD3^+^ T cells. Next, the authors incubated patient PBMC samples with IgG for various time points and measured mean fluorescence intensity of FcγIIIR (CD16^+^) on NK cells. Over the course of 72 h, PBMCs incubated in the absence of IgG had a gradual decrease in FcγIIIR overtime. When cells were incubated with IgG, there was an even greater dose-dependent decrease in FcγIIIR expression.

In an examination of potential differences between IVIg and glucocorticosteroid treatments, Klehmet et al. found that there were distinct differences in the immune cell populations affected by the different treatments [56]. Glucocorticosteroid-treated patients displayed reduced frequencies of NK cells, B cells, and CD4^+^ T cells (i.e., absolute number, naïve, central memory, effector memory, and terminally differentiated CD4^+^ T cells) compared to untreated CIDP patients. On the other hand, IVIg treatment led to reduced frequencies of CD4^+^ effector memory T cells and CD8^+^ central memory and terminally differentiated T cells. Altogether, it is evident that a multitude of immune cell subsets and their secretome are altered in CIDP. More research is needed to establish which one(s) is/are the primary effector(s) so as to design more specific therapeutics.

### Cytokines, chemokines, cell surface, and other soluble factors in immune cells, Schwann cell factors, and the blood nerve barrier (BNB)

#### Cytokines and chemokines

In some patients with CIDP, there are increases in serum IL-2 levels compared to healthy controls and those with other non-demyelinating neurological diseases [57]. Serum TNF-α was also found to be elevated in the active phase in a subgroup of CIDP patients and correlated with clinical severity [58]. Of further note, intense TNF-α staining was evident in macrophages in contact with myelinated fibres while expression of the cytokine was markedly less in cells outside of the nerve [38]. Using in situ hybridization, Mathey and colleagues have also shown that TNF-α and IFN-γ are expressed in CIDP patient nerves within the endoneurium and around epineurial and endoneurial blood vessels by cells that had morphologies similar to that of T cells and macrophages [59]. Within the perineurium, there was strong TNF-α and IFN-γ mRNA expression, and to a lesser extent, IL-2 expression.

In terms of other cytokines and chemokines in sera, Beppu and colleagues analyzed serum cytokine levels in typical CIDP patients, LSS patients (an atypical variant), and healthy controls and found that there was an increase in hepatocyte growth factor (HGF), TNF-α, IL-1β, macrophage inflammatory protein (MIP)-1α, and MIP-1β in CIDP and LSS patients compared to controls [60]. More specifically, typical CIDP patients had elevated levels of MIP-1β compared to controls and elevated HGF compared to LSS patients and controls. In all groups, there was no detectable levels of GM-CSF, IFN-α2, IL-1α, IL-3, IL-5, IL-12(p40), IL-15, or leukemia inhibitory factor [60], but patients in the active phase of CIDP have been found to have elevated levels of serum endothelial leukocyte adhesion molecule-1 (ELAM-1) [61]. Active CIDP patients also display significantly higher production of IL-17 compared to patients in remission and controls, and higher production of IFN-γ and IL-10 compared to controls, while patients in remission also expressed higher levels of IFN-γ than controls.

Cytokines and chemokines have not only been measured in sera. In one study, IL-6 was detected in the CSF but not in the serum of 3/7 CIDP patients [62]. Further, others have shown that in CIDP patient CSF but not the sera, there was an increase in the levels of IL-12 compared to non-inflammatory neurological disorders and healthy controls, but no difference in IL-17 or IL-15 levels [63].

#### Other immunological factors

Cytokines and chemokines are not the only factors altered in immune cells in CIDP. Leppert and colleagues have found that matrix metalloproteinases (MMP)-2 and MMP-9 were upregulated in T cells of CIDP patients compared to non-inflammatory neuropathy controls with no change in the expression of MMP-3 or MMP-7 [64]. However, the upregulation of MMPs in CIDP patient samples was also observed in non-systemic vasculitic neuropathy suggesting that the increase does not contribute directly to the demyelination seen in CIDP.

In addition to MMPs, alterations in signal transducer and activator of transcription (STAT) have been observed. For example, Madia and colleagues showed that p-STAT1, p-STAT3, and T-box transcription factor TBX21 (T-bet) were all highly expressed in CD4^+^ T cells and monocytes of active CIDP patients compared to controls and CIDP patients in remission [65]. In addition, CD8^+^ T cells expressed p-STAT3 more highly in active disease compared to controls and patients in remission.

#### Schwann cell factors

Molecular factors expressed by or secreted by Schwann cells have been explored in the context of CIDP. For example, Murata and Dalakas examined the expression of co-stimulatory molecules and their receptors by Schwann cells [66] and found that BB-1 was expressed on these glial cells. More specifically, BB-1 was expressed by non-myelinating Schwann cells in the various neuropathies examined; however, BB-1 was expressed on the outer layer of myelinating Schwann cells only in the context of CIDP. Of further note, T cells that were in close proximity to the BB-1-expressing Schwann cells stained for CD28 or CTLA-4. Upon further examination, the authors found that the Schwann cells expressing BB-1 also stained for HLA-DR. Finally, in terms of other markers, in a study examining molecules required for T cell activation in CIDP patients’ sural nerve biopsies, it was found that Schwann cells (5/7 patients) expressed the adhesion molecule CD58 (LFA-3) which was not seen in healthy controls [67]. The question is whether Schwann cells are playing a possible antigen presenting cell role in CIDP.

#### BNB

In regard to whether molecular alterations are observed in the BNB in typical and atypical (i.e., LSS and DADS) variants of CIDP, the levels of tight junction proteins produced by immortalized human peripheral nerve microvascular endothelial cells were examined after exposure to patient sera [68]. Cells exposed to patient sera had decreased protein levels of claudin-5 compared to healthy controls, but changes in occludin protein levels were not detected. In keeping with the decrease in claudin-5, transendothelial electrical resistance (TEER) studies revealed significant decreases after cells were exposed to CIDP patient sera. That is, the sera of typical CIDP patients led to the greatest decrease in claudin-5 and TEER values compared to LSS, DADS, or control sera. Further, in comparisons between LSS and DADS, DADS patient sera resulted in lower TEER values than LSS sera but no significant difference in claudin-5 levels. Kanda and colleagues have also identified a decrease in claudin-5 expression in sural nerve biopsies of CIDP patients [69] and additionally noted that there was a decrease in the percent of ZO-1 expressing endoneurial blood vessels compared to other neuropathies.

### Summary of typical CIDP

As evidenced above, typical CIDP is hallmarked by a prominent macrophage and T cell involvement which contributes to demyelination. There is also evidence to suggest that Schwann cells are implicated in typical CIDP. Decreases in Tregs and increases in macrophages may be playing a role in the increased prevalence with age due to the increased accumulation of macrophages with age in the PNS and the discrepancies between the reported age-dependent increases in Tregs and the reported decrease in the cell population under CIDP conditions. The presence of various immune cells is also dependent on the state of the patient whether that be relapsing, remitting, or progressive. For example, in active disease there is a reported increase in Th17 cytokines and cells which declines as the patient transitions into remission. The most common and beneficial treatment for this patient population is IVIg. IVIg has been found to impact T cells, macrophages, B cells, and NK cells to varying degrees.

## Atypical CIDP

In the following section, atypical CIDP variants will be discussed in order of reported prevalence (Table 1).

### Sensory predominant CIDP

In 1992, Oh and colleagues described a group of patients who experienced a monophasic, chronic, progressive, diffuse sensory demyelinating neuropathy [70]. These patients had elevated CSF protein, abnormal pain sensation, and proprioception, and interestingly, reported no motor symptoms. This is intriguing because although the patients only reported sensory abnormalities, they did have motor conduction issues when tested which are indicative of demyelination rather than an axonal neuropathy [70]. Sensory predominant CIDP has been reported to make up between 4 and 35% of CIDP cases [3]. Of further interest, several reports have found cases of patients presenting initially with sensory symptoms and then going on to develop motor symptoms (i.e., weakness) [71, 72]. Patients with sensory predominant CIDP tend to have a younger age of onset relative to other variants of CIDP [73]. Also, these individuals are occasionally misdiagnosed with chronic idiopathic axonal polyneuropathy due to its atypical nature.

#### Chronic immune sensory polyradiculopathy (CISP)

CISP is characterized by chronic, progressive inflammatory demyelinating neuropathy in which the dorsal nerve roots proximal to the dorsal root ganglia (DRG) are targeted resulting in large sensory fibre loss, gait ataxia, and reflex loss [74]. As such, CISP is considered a subtype of sensory predominant CIDP. In addition, these patients have elevated protein in their CSF, enlarged lumbar nerve roots with inflammation, and demyelination.

### Seropositive CIDP

Many studies in recent years have identified patients positive for antibodies against several nodal proteins, specifically neurofascin 155 and 186 (NF155 and NF186, respectively), Gliomedin, contactin 1 (CNTN1), and Caspr which in turn now make up their own clinical variant [75]. In terms of aging in this atypical variant, patients with anti-NF155 antibodies had a significantly younger age of onset compared to patients without antibodies [76–79]. In contrast, those with antibodies against CNTN1 or the CNTN1/Caspr1 complex tended to have an older age of onset [80]. In the CNS, it appears that there is a failure of the attachment of myelin loops with age [81]; this may leave CNTN1 exposed to antibody-mediated attack with increasing age. However, whether or not this occurs in the PNS has yet to be elucidated.

#### Anti-NF155-positive CIDP

The most extensively researched of the seropositive CIDP group is anti-NF155-positive CIDP which makes up 4–18% of CIDP patients [3]. This group of patients tend to have tremors, sensory and cerebellar ataxia, and distal weakness, but the onset of these symptoms is often subacute [75–77, 82, 83]. Patients also tend to have symmetric spinal root and plexus hypertrophy [77]. In people with anti-NF155-positive CIDP, the levels of anti-NF155 seem to fluctuate in conjunction with clinical symptoms [84]; specifically, it appears that patients with higher titers of the antibody present with a more severe disease [85]. Further, some patients with anti-NF155 antibodies may have the IgM variety in contrast to the common IgG4 type that is most commonly seen [86]; although IgG1, IgG2, IgG3, and IgA have also been identified as contributors but to a lesser extent [87]. Interestingly, there is a correlation between presence of antibodies and genetics. That is, the amount of anti-NF155 antibodies in CIDP patients has been associated with an increased likelihood of having a DRB1*15 allele of the HLA class II gene [88]. In this study, 10 out of 13 anti-NF155 antibody-positive CIDP patients possessed the DRB1*15 allele leading the authors to conclude that the allele was a strong risk factor. However, because the allele was not seen in the entire patient pool, it is likely not a necessity in anti-NF155 antibody development.

##### Morphology

In terms of nerve morphology in seropositive anti-NF155 CIDP, no inflammatory infiltrates were seen in sural nerve biopsies in an electron microscopy examination of four CIDP patients with anti-NF155 antibodies [78, 89]. However, there was some paranodal demyelination, detachment of terminal loops, a loss of transverse bands, and a widening of the nodes of Ranvier. In other studies, endoneurial reductions in myelinated fibre density were noted along with a lack of onion bulbs, an absence of macrophage-mediated demyelination, and the presence of edema and myelin ovoids in addition to the aforementioned features [89, 90]. This has led to the conclusion that in this variant there is diffuse demyelination taking place [89]. Manso and colleagues have examined the specific targeting of anti-NF155 IgG4 antibodies and found that they localize to the surface of Schwann cells and Schwann cell microvilli in rat peripheral nerves, but these antibodies do not penetrate into the paranode [91]. In addition, the authors examined the ability of anti-NF155 antibodies isolated from CIDP patients to interfere with the formation of NF155 and CNTN1/Caspr aggregates in vitro and found that the antibodies did not affect clustering. While these antibodies did not affect clustering in vitro, there were effects in paranodal formation in newborn rats prior to postnatal day four. When given anti-NF155 antibodies isolated from CIDP patients prior to the stabilizing of the paranode (i.e., between postnatal day zero and day four), there was a decrease in the levels of NF155 and associated delays and oddities in paranodal formation. When adult rats were given anti-NF155 antibodies daily into the CSF, it led to tail weakness or paralysis as well as ventral root conduction abnormalities which further progressed to hind limb paralysis, gate alterations, and tail paralysis with ongoing days of treatment. Animals treated with these antibodies also showed a decrease in NF155 protein levels in the ventral roots, but not the dorsal roots. From this, the authors concluded that in adult animals, anti-NF155 antibodies may be preventing the maintenance and renewal of paranodes by depleting NF155.

##### Cytokines and chemokines

Cytokine levels have been examined in people with anti-NF155-positive CIDP. In a study examining CIDP patients with and without IgG4 anti-NF155 antibodies and those with non-inflammatory neurological disease, patients’ CSF was examined for various cytokines, chemokines, and growth factors [79]. It was found that CIDP patients with NF155 antibodies had higher levels of CSF protein and of IL-13, IL-8, C–C motif chemokine 11 (CCL11), MCP-1, TNF-α, and IFN-γ compared to non-inflammatory neurological disease patients. They also displayed higher levels of IL-8 and IL-13 and lower levels of IL-1β, IL-1ra, and GM-CSF compared to CIDP patients without NF155 antibodies. Further, when compared to non-inflammatory neurological disease patients, antibody-positive people with CIDP displayed lower levels of IL-1β, IL-1ra, and IL-6. Finally, the CIDP patients without NF155 had higher levels of IFN-γ and lower levels of IL-1ra and IL-4 compared to non-inflammatory neurological disease patients. Based on these findings, the authors concluded that in CIDP patients, there is an increase in Th1 cytokines with the NF155 antibody-possessing group displaying an additional increase in Th2 cytokines.

##### Immune cells

Regarding specific immune cells that may be involved in anti-NF155-positive CIDP, one study was interested in studying autoreactive Th1 cell responses against NF155, NF186, P0 (aa 180-199), and myelin basic protein (MBP) (aa 82-100) in typical CIDP and its variants [92]. Using IFN-γ enzyme-linked immunosorbent assay (ELISA), the authors found more instances of IFN-γ secretion in response to NF155 by PBMCs derived from typical CIDP patients (9/18 patients) compared to other non-immune neuropathy (ON) patients and healthy controls. The latter recorded no responses while activation was noted in 4/9 patients with LSS compared to the ON. A similar pattern was seen for NF186 where LSS had more reactivity (6/9 patients) compared to both ON and healthy controls. As for reactivity to P0, it was found that PBMCs from DADS patients (4/8 patients) differed significantly from the healthy controls, whereas sensory predominant CIDP (12/13 patients) and typical CIDP patients (11/16 patients) were more reactive compared to both healthy controls and the ON group. Samples from LSS (6/9 patients), sensory predominant CIDP (12/13 patients), and typical CIDP patients (10/16 patients) were also significantly more reactive to MBP compared to healthy controls and the ON group, while DADS patient samples were more reactive than only the healthy controls group (4/8 patients). From this, it was concluded that it is common in CIDP patients to have Th1 responses to antigens of the nodal region and from myelin.

#### Anti-contactin1 (CNTN1)-positive CIDP

As we have discussed thus far, much is known about anti-NF155-positive CIDP since it is the most studied of this atypical subtype, but there are other seropositive CIDP conditions that display other antibodies besides anti-NF155. CNTN1 is the binding partner of NF155. Anti-CNTN1 antibodies have been identified in some CIDP patients [75, 93] with the most prevalent subtypes being IgG3 and IgG4 [93]. Others have reported that this particular seropositive subtype makes up 1–7% of CIDP patients [3]. In a study by Doppler and colleagues, they identified four anti-CNTN1-positive patients and noted that all of the patients had been initially misdiagnosed with Guillain-Barré Syndrome due to the rapid and aggressive onset of the symptoms [93]. Interestingly, patients positive for these antibodies predominantly displayed motor symptoms [94], sensory ataxia [95], weakness, and occasionally tremors, that followed a relapsing-remitting disease course [75, 93]. These CIDP patients also had an older age of onset compared to non-seropositive CIDP patients [75, 94]. On a histological level, this patient group displayed high numbers of endoneurial macrophages, but a typical number of T cells. There was also axonal degeneration, but no onion bulbs and little incidence of demyelination. Skin biopsies showed widening of nodes of Ranvier and a loss of immunohistochemical staining for Caspr and/or neurofascin in several of the patients. Some of these findings were also observed by Querol and colleagues who identified patients that were positive for antibodies against CNTN1 or the CNTN1/Caspr1 complex but not against Caspr alone although this has been reported by other groups [94].

Towards understanding the prevalence of anti-CNTN1 antibodies in CIDP patients, Mathey and colleagues performed ELISA on sera from 44 CIDP patients and found that three patients had anti-NF155 antibodies and a further three had anti-CNTN1 antibodies primarily of the IgG4 type [96]. In both of these groups, patients with one antibody did not have the other (e.g., NF155 positive, CNTN1 negative, and vice versa). The detection of anti-CNTN1 IgG4 antibodies is noteworthy since these antibodies have been shown to mitigate cell aggregation typically seen via the formation of the Caspr/CNTN1/NF155 complex. As a consequence, defects arise in the nodal regions because of specific targeting of the immunoglobulin domains on CNTN1 in an *N*-glycosylation-dependent manner [97].

In confirmation of this targeting, Manso and colleagues have examined the impact of IgG1 and IgG4 anti-CNTN1 antibodies isolated from CIDP patients on nerve structure [98]. They found that IgG4 isotype antibodies have the ability to enter into the paranode. In a passive transfer experiment in rats, the authors immunized the animals with P2 protein and after 12, 19, 26, and 33 days, they began giving weekly injections of anti-CNTN1 antibodies. Progressively, the rats displayed worsening clinical symptoms and upon sacrifice, the rats had tail paralysis and gait abnormalities. Further investigation revealed that the antibodies were able to penetrate into the paranodes and disrupt the CNTN1/Caspr/NF155 complex. It was also noted that there was a selectivity for the ventral root axons and axons of a smaller diameter as reflected in abnormalities in ventral root conduction studies.

#### Anti-NF186-positive and anti-gliomedin-positive CIDP

In the aforementioned study by Mathey and colleagues, the authors noted that NF186 or gliomedin antibodies were not detected in the sera of their CIDP patient pool [96]. However, Devaux et al. found that 30% of CIDP patient serum did label the nodal region and in a cell binding assay found that 24% of the CIDP patient samples had antibodies against NF186, gliomedin, neuronal cell adhesion molecule (NrCAM), and/or CNTN1 with 16% having reactivity to just one and 8% having reactivity against more than one antigen [99]. Delmont and colleagues [100] also identified a subpopulation of CIDP patients who possessed anti-NF140/186 antibodies that made up 2% of their 246 CIDP patient pool. These patients had a distinct phenotype characterized by subacute onset, sensory ataxia, and in some instances, cranial nerve involvement. Further, unlike patients with NF155 antibodies, this group did not have tremors or pain, and often had a comorbid autoimmune disorder. Of note, this group showed a positive response to IVIg and steroid treatment. It is possible that the diverse results may be due to sample size and patient demographics.

#### Anti-Caspr-positive CIDP

In a handful of cases, some CIDP patients were positive for anti-Caspr antibodies [75, 101]. It has been reported that 1–3% of CIDP patients may have these antibodies [3]. This very small group of patients presented with pain and many of the other symptoms seen in the other seropositive CIDP patients; however, they did not have tremor or ataxia. The onset of symptoms was subacute and severe with a motor dominant presentation.

#### Other antibodies in CIDP

Aside from the previously mentioned nodal antibodies found in some atypical CIDP patients, other groups have reported the presence of other antibodies. Terryberry and colleagues identified neurofilament heavy (NF-H) subunit autoantibodies in 88% (15/17) of their CIDP patient serum samples and anti-tubulin antibodies in 24% (4/17) of patient samples [102]. Further, Kuwahara and colleagues have additionally reported a small group of CIDP patients presenting with antibodies against gangliosides, specifically, anti-LM1, anti-GM1/LM1, and/or anti-GD1b/LM1 antibodies [103]. In this small group, patients did not have damage to the cranial nerves and were more likely to present with ataxia. The age of the patients presenting with these antibodies were also significantly older than that of other CIDP patients.

##### Target and function

In order to elucidate what the antibodies may be binding to or what their roles may be, Kwa and colleagues examined reactivity of CIDP patient sera against cultured human Schwann cells and found that 26% of CIDP patients displayed staining against the leading edge and external processes of Schwann cells [104]. The authors also examined reactivity to neurons (i.e., differentiated human teratocarcinoma hNT2 neurons) and found that patient sera bound to the neurite growth cones. These findings were validated on teased mouse nerve fibres where CIDP patient sera bound in all instances. The authors next attempted to determine the targeted epitope and demonstrated that it was *not* p75^LNTR^, growth-associated protein (GAP)-43, or NDRG3 which are typically found on the leading edge of Schwann cell processes.

In another study by Allen and colleagues, the prevalence of antibodies against peripheral myelin proteins in CIDP patient sera was examined [105]. They found that 25% (8/32) of CIDP patients in the study possessed the antibodies which were significantly more than in the control group. Notably, 6 out of the 8-antibody-possessing CIDP patients appeared to have antibodies against P0. Yan and colleagues also found that some CIDP patients (6/21) possessed serum anti-P0 IgG antibodies [106], and when this serum was given to animals, it led to demyelination which was not seen when P0 protein was added to the sera prior to transfer into experimental animals. However, unlike Allen et al. and Yan et al., Inglis and colleagues did not observe any significant differences in reactivity to P0 or P2 peptide sequences in CIDP patient sera [107]. In alignment with Inglis et al., Sanvito and colleagues also reported low frequencies of antibodies against peripheral myelin proteins in CIDP patients and controls [108]. It would be interesting to test whether the disparate findings are related to the P0 peptide sequence being assessed. Finally, aside from P0, antibodies against other potential peripheral nerve antigens have been found such as PMP-22 where anti-PMP-22 antibodies were detected in the serum of 35% of CIDP patients [109].

##### Therapy

Altogether, seropositive CIDP patients typically have a poor response to IVIg and/or steroid treatments which are beneficial in other CIDP subtypes. However, seropositive CIDP patients tend to have a positive result when treated with rituximab [110, 111]. Rituximab is an antibody that targets CD20 on B cells [112], which is in keeping with studies showing reductions in antibody levels when treated with the drug [110].

### Lewis-Sumner Syndrome (LSS)/multifocal acquired demyelinating sensory and motor neuropathy (MADSAM)

In 1982, Lewis and colleagues highlighted several cases of asymmetric multifocal neuropathy [113]. It has been reported that 8–15% of CIDP patients may have this variant [3]. These patients demonstrated multifocal conduction block on electrophysiologic tests and biopsies revealed that there were many instances of focal demyelination and areas of remyelination. Both sensory and motor fibres were affected with a subacute onset. Similar to other CIDP patients, those affected by LSS have elevated protein levels in their CSF [114]. Interestingly, biopsies from the patients have revealed little to no inflammation in the perineurium or epineurium. Beppu and colleagues have noted that LSS patients displayed higher amounts of TNF-α, IL-1β, and MIP-1α in their serum compared to healthy controls [60].

### Motor predominant CIDP

Patients with motor predominant CIDP tend to present with symmetrical upper limb weakness that initially involves the cervical nerve root and brachial plexus and then progresses to generalized areflexia [115]. The variant is reported to make up 4–10% of CIDP cases [3]. The course of the disease is typically relapsing-remitting and IVIg treatment can be beneficial. As reported by Kimura and colleagues, within the first 2 years of disease the patients experience frequent relapses that taper off as a rarity thereafter. In addition to the aforementioned features, Sabatelli and colleagues additionally reported that motor predominant CIDP patients have electrophysiological abnormalities that were only detected in motor fibers [116]. Moreover, symptoms were still restricted to motor functions after follow-ups with the patients. In regard to immune cells, Mei and colleagues examined a group of CIDP patients, all of which presented with dominant motor involvement, and reported an upregulation of IL-17, IL-8, and IL-6 prior to the beginning of IVIg treatment and a downregulation of IL-4, IL-5, and IL-7 (Th2 cytokines) after an examination of 16 cytokines in CIDP patient CSF [117]. Following IVIg, the CIDP patients maintained IL-8 upregulation and downregulation of IL-5 and IL-7 in the CSF.

### Distal acquired demyelinating symmetric neuropathy (DADS)

As its name implies, DADS patients present with symmetric, distal, primarily sensory impairment [118]. DADS has often been referred to as an atypical variant of CIDP; however, two thirds of these patients have IgMκ monoclonal gammopathies. In addition, a large portion of DADS patients possess anti-myelin-associated glycoprotein (MAG) antibodies. As distinguished by Larue and colleagues [119], it is possible that DADS patients who do not have antibodies against MAG are considered a subtype of CIDP; this is reflected in the EFNS/PNS CIDP diagnosis guidelines [120]. This variant is believed to encompass 2–10% of CIDP cases [3].

## Major animal models of CIDP

### Chronic experimental autoimmune neuritis (EAN): a relapsing-remitting disease model

EAN is frequently used as a model to study Guillain-Barré Syndrome; however, many studies have used the model for CIDP experiments because of the relapsing-remitting nature of EAN in the chronic phase. EAN is induced by immunizing rodents with whole myelin or specific myelin proteins [121]. EAN can also be induced through the adoptive transfer of T cells sensitized to P2 myelin antigen [122]. A study using this model to examine CIDP-like pathology has demonstrated that Lewis rats immunized to develop EAN and treated with intranasal IL-17 for six days following disease onset, had worse disease at peak in a dose-dependent manner; however, they had a shorter disease duration than untreated EAN rats [123]. In the initial phase in the treated group, there was an intense immune infiltration consisting of CD4^+^ and CD8^+^ T cells and macrophages, and an increased expression of MHC class II compared to controls, thus mimicking some aspects of the human disease.

Ng and colleagues have also demonstrated that pan-neurofascin antibodies lead to worse disease and prolonged symptoms in chronic EAN, but it was not sufficient to drive the disorder when the animals were not immunized with P2 peptide in disease induction [87]. Similar findings were obtained by Yan and colleagues who gave either a pan-neurofascin monoclonal antibody or IgG_2a_ control at EAN symptom onset [124]. They found that antibody-treated animals had significantly worse disease after 48 h post-injection. Further, the antibody-treated animals recovered by day 18 after disease induction whereas controls only recovered at day 15. It will be interesting to see if this model can lead to the discovery of treatments that are more specific than IVIg.

### Spontaneous autoimmune peripheral polyneuropathy (SAPP): a progressive disease model

In 2001, Salomon and colleagues developed a mouse model that more accurately mimics the clinical phenotype seen in progressive CIDP [125]—spontaneous autoimmune peripheral polyneuropathy (SAPP). The model is derived in non-obese diabetic (NOD) mice that have been manipulated to be deficient in the co-stimulatory molecule B7-2 (CD86). Originally, SAPP was designed to determine the role of B7-2 in type 1 diabetes mellitus. However, upon examination, the researchers found that the mice did not develop diabetes; instead, they displayed progressive symmetrical limb paralysis beginning in the hind limbs that eventually affected front limbs. In normal NOD mice treated with monoclonal anti-B7-2 antibodies, one third of the treated mice developed the same CIDP-like phenotype which did not occur in control or phosphate-buffered saline-treated mice. SAPP-developing mice presented with inflammatory infiltrates in their dorsal and ventral spinal roots, DRG, and nerves. Further, peripheral nerves of these mice displayed extensive demyelination, irregular myelin thickness, and many instances of irregular morphology at the nodes of Ranvier. Dendritic cells, CD4^+^ and CD8^+^ T cells were revealed to be infiltrating into the nerves of SAPP mice with the co-stimulatory molecule B7-1 being expressed on CD11b^+^ and CD11c^+^ cells. Of note, SAPP is mediated by antigen-specific T cells; this was shown through adoptive transfer studies of reactive T cells from SAPP mice which led to the development of SAPP in recipients, but this was not seen when T cells were transferred from typical NOD mice. In a further experiment, the authors demonstrated that the initiation of SAPP was dependent on CD4^+^ T cells but they did not rule out the contribution of CD8^+^ T cells in further disease pathogenesis.

#### Morphology

Ubogu and colleagues have studied the disease course and nerve morphology of SAPP in detail and found that initially there is focal demyelination with mononuclear cell infiltration [126]. At peak disease severity, there was axonal loss associated with the inflammatory infiltrate (which was revealed to be mainly composed of macrophages and to a lesser extent CD3^+^ T cells and CD19^+^ B cells), and more diffuse instances of demyelination within the nerves of these mice. As SAPP progresses, onion bulbs start to form due to cyclical demyelination and remyelination and the immune cell infiltration is associated with endoneurial edema. These SAPP-developing mice also demonstrated a loss in S100β and NF-H immunohistochemical staining compared to controls.

#### Tregs and Bregs

Quan and colleagues have gone on to more closely examine the role of various immune cells in SAPP particularly regulatory T (CD4^+^ Tregs) and B cells (CD19^+^CD1d^hi^CD5^+^) [127]. They determined that in the spleens and lymph nodes of female SAPP-developing mice, there was a decrease in the number of both Tregs and Bregs. In males however, there was a decrease in the number of Tregs in the lymph nodes but not the spleen, and a decrease in Bregs in both the spleen and the lymph nodes. Tregs from both normal NOD and SAPP-developing mice at 3 months of age were able to inhibit effector T cell proliferation in response to antigens while Bregs had no impact. When examining the functionality of these cells in culture, the authors found that Tregs from normal NOD and SAPP-developing mice did not impact CD4^+^IFN-γ- or CD4^+^IL-17-producing cells, but there was an increase in CD4^+^ IL-10-producing cells. The same effect was seen in co-cultures with Bregs, although in Tregs and Bregs from SAPP-developing B7-2^-/-^ mice, the effect was not as pronounced. Furthermore, the authors performed an adoptive transfer experiment using Tregs and Bregs from normal NOD mice and transferred them to SAPP-developing mice. They found that the transfer of Tregs at disease onset was sufficient to attenuate disease severity; however, this was not the case with Bregs. Because of this, the authors performed the adoptive transfer of Bregs prior to disease onset. Transferred Bregs prior to disease onset led to a significantly weakened disease severity. Both the adoptive transfer of Tregs or Bregs led to a decrease in splenocyte proliferation, increase in B10 cells in the spleen and lymph nodes, increase in Tregs in the spleen, and increase in CD4^+^IL-10^+^ T cells in the spleen and lymph nodes. Next, to determine the role of B cells in SAPP, the authors used a mutant *B7-2*^*-/-*^ mouse that is also null for mature B cells. In the absence of mature B cells, SAPP did not develop. In addition, these mutant mice had increased percentages of Tregs in the spleen and lymph nodes compared to typical SAPP-developing mice. Further, Tregs from the mutant mice had a slightly better ability to induce CD4^+^IL-10^+^ T cells in culture. Thus, Tregs and Bregs may have the potential to reduce inflammation in CIDP.

#### Dendritic cells

The role of dendritic cells in SAPP has also been examined [128]. In the lymph nodes of SAPP mice, there is an increase in the percentage of CD11b^+^ dendritic cells at 8 months of age compared to normal NOD mice. In the spleens of SAPP-developing mice at 2 months of age, there was an increased percentage of MHC class II^+^, CD40^+^, ICOSL^+^, and B7-1^+^CD11b^+^ dendritic cells and of B7-1^+^CD11b^+^ and B7-1^+^CD8α^+^ dendritic cells at 8 months of age. Next, to assess the ability of dendric cells to capture antigen, the authors developed a fluorophore-tagged P0 extracellular domain and injected it into SAPP mice. In the SAPP mice, there was a decrease in fluorescently-labeled CD11b^+^ and CD11b^-^ dendritic cells compared to normal NOD mice. Further, the authors examined CD4^+^ T cell proliferation in response to CD11b^+^ dendritic cells and found that dendritic cells from SAPP mice were less able to stimulate T cell proliferation compared to normal NOD mice. Using RT-PCR and ELISA, the authors assessed cytokine levels in unstimulated dendritic cells from normal NOD mice and SAPP-developing mice and found that there was a decrease in IL-10 production and secretion in the SAPP-derived dendritic cells compared to normal NOD mice. The authors then used a mouse where CD4^+^FoxP3^+^ T cells were tagged with eGFP in order to assess the ability of SAPP mice to generate Tregs, and it was determined that in SAPP mice, there was decreased proliferation of Tregs. Next, the authors performed adoptive transfer studies with P0-pulsed dendritic cells from normal NOD or SAPP-developing mice into SAPP-developing mice. They found that adoptive transfer of dendritic cells from normal NOD mice significantly increased tolerance to the development of SAPP. Upon evaluation, the mice that developed tolerance had decreased splenocyte proliferation induced by P0 or its extracellular domain, a decrease in CD4^+^TNF-α^+^ T cells in the spleen, an increase in CD4^+^IL-10^+^ T cells in the spleen and lymph nodes, and an increase in Tregs in the spleen. Because of the decrease in IL-10 seen in the SAPP-developing mice, the authors examined whether that was contributing to the lack of tolerance development following adoptive transfer of their dendritic cells. When dendritic cells were conditioned with IL-10 prior to adoptive transfer, it was sufficient to induce tolerance in SAPP-developing mice and was able to convert SAPP-developing dendritic cells into a normal NOD mouse dendritic cell phenotype.

#### Antigen(s)

In terms of the antigen(s) being targeted, Louvet and colleagues developed antigen-specific T cell hybridomas from SAPP-developing mice and determined that P0 was the main protein that these hybridomas were reactive against [129]. Next, they generated a P0-specific T cell receptor transgenic mouse (POT). In these POT animals, there was a decrease in the percentage of CD4^+^ and CD8^+^ T cells in the thymus and an increase in the CD4/CD8 ratio. In addition, proliferation of T cells in POT animals was evident but not in the normal NOD mice. However, the POT mice did not develop SAPP and it was only when POT mice were crossed with a RAGKO mouse line that is deficient in T cells, that the mice developed a rapid and severe form of SAPP with extreme weight loss and death within 3 to 5 weeks of age. These mice also had a prominent immune infiltrate and demyelination. Further, adoptive transfer of splenocytes from the NOD-POT-RAGKO mice led to neuropathy in immunodeficient NOD mice.

In regard to molecular mediators in immune cells that may be involved in SAPP pathogenesis and/or disease resolution, it was shown that in the spleens of SAPP mice, prior to disease onset at 4 months of age, there is a decrease in IL-10 and MCP-1 and an increase in IL-17 mRNA [130]. IL-17 mRNA then declines to baseline levels as the clinical phase of the disease begins around 8 months of age. In the sciatic nerve at 8 months of age, there is an upregulation of TNF-α, IFN-γ, IFN-γR, C-X-C motif chemokine ligand 10 (CXCL10), and regulated on activation, normal T cell expressed and secreted (RANTES) mRNA, and to a lesser extent, an upregulation of MIP-1α, MIP-1β, CXCL16, and MCP-1. Interestingly, there was no upregulation of IL-17 in the sciatic nerves of SAPP mice as seen in the spleen. From this, it was concluded that CXCL10 and RANTES, both being Th1 cytokines, are critically involved in SAPP development. In this study, the authors also determined that P0 was the target autoantigen in SAPP since their splenocytes produce IL-2 and IFN-γ in response to P0. Based on the cytokine and chemokine data as well as the splenocyte production of IFN-γ, the authors concluded that IFN-γ is critical for mediating nerve damage in SAPP.

As to what the initiating factor(s) may be in SAPP, Su and colleagues demonstrated that in NOD mice, a point mutation on the autoimmune regulator gene (Aire) contributes to the development of SAPP [131]. In their model, the mice developed progressively ascending bilateral limb weakness that was caused by intense immune infiltration into the nerves composed of CD4^+^ Th cells and macrophages. The mice also had many instances of focal demyelination and unlike the original SAPP model, the mice with the Aire mutation developed diabetes with the same incidence rate as normal NOD mice. This model of SAPP can be initiated through the adoptive transfer of CD4^+^ Th cells from the Aire-mutated NOD mice into immunodeficient NOD mice. Interestingly, CD8^+^ T cells were also able to induce SAPP but at a much lesser frequency. The CD4^+^ T cells infiltrating the nerves of the Aire-mutated NOD mice were found to produce IFN-γ in 40% of instances which would be indicative of a Th1 effector response, and very minimal IL-4- and IL-17-producing cells. The authors next determined that P0 was the target PNS autoantigen regulated by the Aire gene, and this was the product of defective negative selection of T cells specific for P0 in the thymus. In keeping with these findings, others have shown that mice heterozygous for P0 also develop a CIDP-like phenotype characterized by multi-focal demyelination (particularly of motor fibres) with some axonal loss, endoneurial edema, and macrophage and lymphocyte accumulation around motor nerve roots [39].

The extracellular matrix protein called periostin that is produced by Schwann cells may be another initiating antigen in SAPP [132]. The protein was only seen in the perineurium of control mice; however in the SAPP mice, the protein was diffuse in the endoneurium. The authors went on to show that when mice are deficient in the protein, there is a delay in the onset of SAPP and a reduction in recruited T cells and macrophages. Furthermore, it was determined that periostin expressed by Schwann cells was critical for the timely recruitment of macrophages but not T cells and that this response is dependent on the periostin receptors Integrin Subunit Alpha V (ITGAV) and CD11b (i.e., ITGAM). It was also shown that the macrophages were the pathological driving force of the neuropathy because when they were depleted, there was a delay in disease onset and a reduction in severity. In addition, the authors confirmed the presence of periostin in two of five CIDP patient nerve biopsies via immunostaining whereas none was detected in axonal neuropathy controls. Towards elucidating the mechanistic pathway, they cultured human Schwann cells in neuregulin 1, TGF-β, or both and found an increase in periostin expression in the TGF-β or both TGF-β and neuregulin 1 condition. As such, the authors speculated that early infiltrating CD4^+^ T cells may be responsible for initiating SAPP by secreting TGF-β and thus causing an increase in periostin in Schwann cells. This would drive recruitment of macrophages which then leads to a positive feedback relationship between macrophages and T cells.

Altogether, it is heartening to see that the models of CIDP have been evolving closer to the human condition because of the increasing amounts of data being produced in the field. This will only increase the opportunity for more selective therapies being developed for CIDP.

## Conclusion

CIDP is a highly variable condition with certain variants being associated with older ages of onset and immune profiles. Due to the contributions of aging immune cells such as T cells and macrophages, this may play a role in the lessened frequency of functional recovery from CIDP in older patients. Therefore, more studies need to be conducted to specifically examine the age-related changes in immune cells that may be contributing to CIDP.

## Data Availability

Not applicable

## Abbreviations

BAFF: B cell activating factor
BDNF: Brain-derived neurotrophic factor
BNB: Blood nerve barrier
Caspr: Contactin associated protein 1
CCL11: C–C motif chemokine 11
CIDP: Chronic inflammatory demyelinating polyradiculoneuropathy
CISP: Chronic immune sensory polyradiculopathy
CNTN1: Contactin 1
CSF: Cerebrospinal fluid
CXCL10: C–X–C motif chemokine ligand 10
DADS: Distal Acquired Demyelinating Symmetric Neuropathy
DRG: Dorsal root ganglia
EAN: Experimental autoimmune neuritis
ELAM-1: Endothelial leukocyte adhesion molecule - 1
ELISA: Enzyme-linked immunosorbent assay
GAP-43: Growth associated protein - 43
GDNF: Glial cell line-derived neurotrophic factor
GM-CSF: Granulocyte-macrophage colony-stimulating factor
HGF: Hepatocyte growth factor
HLA: Human leukocyte antigen
HPRT: Hypoxanthine-guanine phosphoribosyltransferase
IFN: Interferon
IL: Interleukin
ITGAV: Integrin subunit alpha V
IVIg: Intravenous immunoglobulin
LSS: Lewis-Sumner syndrome
MADSAM: Multifocal acquired demyelinating sensory and motor polyneuropathy
MAG: Myelin-associated glycoprotein
MBP: Myelin basic protein
MCP-1: Monocyte chemoattractant protein - 1
M-CSF: Macrophage colony-stimulating factor
MHC: Major histocompatibility complex
MIP: Macrophage inflammatory protein
MMP: Matrix metalloproteinases
Nav: Voltage-gated sodium channel
NF155: Neurofascin 155
NF186: Neurofascin 186
NF-H: Neurofilament heavy subunit
NGF: Nerve growth factor
NK: Natural killer
NOD: Non-obese diabetic
NrCAM: Neuronal cell adhesion molecule
ON: Other non-immune neuropathy
P0: Myelin protein zero
PBMC: Peripheral blood mononuclear cell
PMP-22: Peripheral myelin protein 22
PNS: Peripheral nervous system
POT: P0-specific T cell receptor transgenic
RANTES: Regulated on Activation, Normal T Cell Expressed and Secreted
SAPP: Spontaneous autoimmune peripheral polyneuropathy
STAT: Signal transducer and activator of transcription
T-bet: T-box transcription factor TBX21
TEER: Transendothelial electrical resistance
Th: T helper
TNF: Tumor necrosis factor
Treg: Regulatory T cell
TSAd: T cell specific adapter protein
